# Proteolysis of proBDNF Is a Key Regulator in the Formation of Memory

**DOI:** 10.1371/journal.pone.0003248

**Published:** 2008-09-24

**Authors:** Philip Barnes, Kerrie L. Thomas

**Affiliations:** Cardiff School of Biosciences, Cardiff University, Cardiff, United Kingdom; University of Parma, Italy

## Abstract

It is essential to understand the molecular processes underlying long-term memory to provide therapeutic targets of aberrant memory that produce pathological behaviour in humans. Under conditions of recall, fully-consolidated memories can undergo reconsolidation or extinction. These retrieval-mediated memory processes may rely on distinct molecular processes. The cellular mechanisms initiating the signature molecular events are not known. Using infusions of protein synthesis inhibitors, antisense oligonucleotide targeting brain-derived neurotrophic factor (BDNF) mRNA or tPA-STOP (an inhibitor of the proteolysis of BDNF protein) into the hippocampus of the awake rat, we show that acquisition and extinction of contextual fear memory depended on the increased and decreased proteolysis of proBDNF (precursor BDNF) in the hippocampus, respectively. Conditions of retrieval that are known to initiate the reconsolidation of contextual fear memory, a BDNF-independent memory process, were not correlated with altered proBDNF cleavage. Thus, the processing of BDNF was associated with the acquisition of new information and the updating of information about a salient stimulus. Furthermore, the differential requirement for the processing of proBDNF by tPA in distinct memory processes suggest that the molecular events actively engaged to support the storage and/or the successful retrieval of memory depends on the integration of ongoing experience with past learning.

## Introduction

Transcriptional and post-translational molecular events are required for the consolidation of information into long-term memories and are thought to lead to the synaptic structural changes that maintain the memory [1], [2]. Originally described by Pavlov (1927), extinction occurs when a conditioned stimulus (CS) is presented without reinforcement of a biologically salient unconditioned stimulus (US), manifesting as a weakening of the conditioned response. Although historically extinction has been viewed as unlearning [3], [4], 5, it is currently viewed as the generation of a new memory about a CS [6]. The extinction memory competes with the original memory for control of behaviour. The protein synthesis-dependent nature of extinction [7] perhaps further emphasises that extinction is a long-lasting memory that is independently acquired and stored.

The molecular mechanisms underlying the formation of long-term fear memory [8], [9], [10] share a remarkable similarity with those required for the primary model of memory formation in neuronal circuits, long-term-potentiation [11]. The activation of these particular molecules may contribute to the enhancement of synaptic strength in the hippocampus and amygdala observed upon the encoding of fear memory [12], [13]. Similar plasticity-related molecular processes maybe required for consolidation and extinction [14], [15], [16], [17]. However, reports indicating that activation of CB1, calcineurin and PI3K-dependent signalling pathways are selectively required for the extinction of fear memory [18], [19], [20], not only suggest that the molecular processes of extinction dissociate from those of consolidation but may more closely correlate with the plasticity processes of long-term depression or depotentiation [21], [22], [23].

We have recently shown *in vivo* that the activity of the secreted neurotrophin, brain-derived neurotrophic factor (BDNF) in the hippocampus is required for the consolidation of hippocampal-dependent contextual fear memory [24]. We also showed that reconsolidation, the restabilisation of the labile memory following the recall by a brief exposure to a reminder stimulus, was not dependent on BDNF. More specifically, we showed that consolidation was critically dependent on the mature form of the neurotrophin, mBDNF. mBDNF is generated by the proteolytic cleavage of the precursor, proBDNF, by protease tissue plasminogen activator (tPA)-mediated activation of plasmin [25], [26]. Studies in *in vitro* preparations have compellingly shown the requirements for mBDNF and proBDNF for hippocampal LTP and LTD respectively [27], [28]. Here using a strategy of independently manipulating two fear memories in the same animal, and using temporally and regionally restricted manipulations of BDNF levels, we show that the processing of proBDNF is positively correlated with the acquisition but negatively correlated with extinction.

## Materials and Methods

### Subjects

The subjects were adult male Lister hooded rats weighing 280–350 g. They were housed in pairs, in holding rooms maintained at 21°C on a reversed-light cycle (12 h light/dark; lights on at 10:00 P.M.). All experiments were conducted in the dark period of the rats. Food and water were freely available throughout the experiment. All procedures were conducted in accordance with local Cardiff University Ethical Committee approval and the United Kingdom 1986 Animals (Scientific Procedures) Act (Project license PPL 30/2236).

#### Surgery, ODN, ANI and tPA STOP infusions, and histological assessment of cannula placement

Performed as described by [24] with the exception that the rats were anaesthetised using isoflurane [flow rates: -oxygen; 0.8 liter/minute, NO_2_; 0.4 litre/minute] and were implanted with stainless steel double guide cannulae (Plastics One, 22 gauge, 3.8 mm centre-to centre, 3 mm below pedestal) aimed at the dorsal hippocampus (AP -3.50, relative to bregma). Stainless steel double cannulae 1 mm longer than guide cannulae was inserted into the guide cannulae to maintain patency during recovery. Subsequent histological analysis revealed accurate placements in all cannula-implanted rats (data not shown). Infusions were carried out using a syringe pump, connected to injectors (28 gauge, projecting 1 mm beyond the guide cannulae) by polyethylene tubing. ODNs were PAGE-purified phosphorothioate end-capped 18-mer sequences, resuspended in sterile PBS to a concentration of 1 nmol/µl: BDNF antisense ODN, ASO, 5′-TCT TCC CCT TTT AAT GGT-3′; BDNF missense ODN, MSO, 5′-ATA CTT TCT GTT CTT GCC-3′. All ODN sequences were subjected to a BLAST search on the National Center for Biotechnology Information BLAST server using the Genbank database. Antisense sequences had positive matches only for their target mRNA sequences, and no other rat or human coding sequences. Control missense sequences, which included the same 18 nucleotides as the ASO but in a scrambled order, did not generate any full matches to identified gene sequences in the database. Anisomycin (ANI, 80 mg/ml, Sigma) and tPA-STOP (4 mM, ADI) was dissolved in sterile PBS. The PBS vehicle was used for habituation infusions in all rats 1 day before conditioning and to act as control infusions on the day(s) of training. ODNs (1.0 µl per side; 0.125 µl/min) or tPA-STOP (1.5 µl per side; 0.125 µl/min) were infused 90 min prior to conditioning or context reexposure, and ANI (1.0 µl per side; 0.5 µl/min) were infused immediately after context reexposure.

On completion of the behavioural testing, the rats were killed by CO_2_ asphyxiation, and the brains removed and fixed in 4% fresh paraformaldehyde in 0.1 M phosphate buffered saline for at least 48 hours before being transferred to 20% sucrose in PBS solution for cryoprotection. Forty-micrometer coronal sections through the dorsal hippocampus were cut on a freezing microtome, mounted onto gelatine-coated slides and Nissl-stained with thionin. The sections were examined under a light microscope and the subjects were only included if the infusion cannulae tracts terminated bilaterally in the hippocampus and there was no damage to adjacent brain structure or gross ventricular enlargement. Subsequent histological analysis revealed accurate placements in all cannula-implanted rats (data not shown).

#### SDS-PAGE and Western Blotting

Following fear conditioning/retrieval test rats were sacrificed by carbon dioxide inhalation. The rats were decapitated and the brain was rapidly removed and placed on ice. The hippocampal dentate gyrus/CA3 and CA1 regions were microdissected and frozen on dry ice prior to storage at −80°C. Tissue lysates and Western blotting were performed essentially as previously described [24]. Proteins (4–10 µg) were separated on 16.5% Tris-Tricine gels at a constant voltage of 80 V and then transferred to Hybond-P PDVP membranes (Amersham Biosciences) at a constant voltage of 100 V for 1 hr. Blots were blocked in 5% non-fat in 0.01 M Tris-buffered saline solution containing 1% Tween 20 (TBST), and this TBST solution was used for all subsequent washes. Primary and secondary antibodies were diluted in TBST containing 0.5% Tween 20 and were used at the following concentrations: Arc (H-300 Santa Cruz), 1∶10000; BDNF (AP1779SP, Chemicon), 0.1 µg/ml, β-actin (AbCam), 1∶20 000; goat anti-rabbit and goat anti-mouse IgG (whole-molecule)-peroxidase conjugates (Sigma), 1∶10 000). Blots were developed using ECL Advance detection (Amersham Biosciences) and opposed to autoradiographic film. Autoradiographs of each Westerns blot were developed to be linear in the range used for densitometry for each protein target and for β-actin. Autoradigraphic images were captured on a Sharp JX330 Scanner using Labscan v2.0 (Pharmacia Biotech) and quantified using ImageMaster 1D Prime v.3.0 (Amersham Pharmacia Biotech). For analysis, optical density (OD) values and the band areas were obtained for each microdissected hippocampal sample for both the target protein (Arc/Arg3.1, BDNF) and the β-actin loading control to derive an amount figure. Averaging the amount of β-actin across samples on each Western blot and deriving a normalization factor for each sample corrected loading variation.

#### Contextual Fear Conditioning in Two Contexts

Each rat received two conditioning trials in two different contexts separated by 24 hours. Individually, rats were first pre-exposed for 3 d to two experimental chambers (contexts) for 20 min/d. These contexts were designed to differ in a number of features including size, spatial location, odor, and lighting. In addition, to further distinguish the two contexts, exposure to each chamber was separated by a minimum of 4 hours. The first conditioning trial was given 24 hours later. Conditioning consisted of the rats being placed individually in a chamber for 3 min. After 2 min a single scrambled footshock (0.5 mA for 2 s) was delivered. After 24 hours the rats were returned to the other conditioning chamber for 3 min and they received a single scrambled footshock (0.5 mA for 2 s) after 2 min. The order of the contexts that the rats were conditioned to was counterbalanced in each experiment. *Extinction training*: Each rat received two extinction training trails in the two different conditioned contexts separated by 24 hours. One or two days after contextual fear conditioning, rats were re-exposed to one of the conditioned contexts either for 2 or 10 min. 24 hours later the rats were exposed to the other conditioned training contexts for either 2 min or 10 min. The order of the conditioned contexts that the rats were exposed to during extinction training was counterbalanced. *Retrieval tests*: Four or five days, and sometimes 14 days, later each rat was given two contextual fear memory retrieval tests (T1 and T2, respectively) separated by 24 hours. The rats were placed into one of the conditioned contexts for 2 min and the following day they were exposed for 2 min to the other conditioned context. The order to which each rat was exposed to the two contexts during the retrieval trails was the same as during the conditioning training.

#### Contextual Fear Conditioning in a Single Context

Where indicated, rats were habituated to handling by placing them for 20 min in one of two distinct conditioning contexts for 3 d (for details see above), the final habituation session preceding conditioning by 24 hrs. During a 3 min conditioning training trial, rats received a single scrambled footshock (0.5 mA for 2 s) 2 min after being placed into the conditioning context. Extinction training 3 d later consisted of exposing rats to the conditioned context for either 2 min or 10 min.

#### Analysis and Statistics

Freezing behavior served as a measure of conditioned fear to the contexts during the conditioning, extinction training and retrieval tests of the behavioural procedures. This was video-recorded and quantified by an observer blind to the experimental group. One unit of freezing was defined as a continuous absence of movement other than that required for respiration in 1 s sampled every 10 s. Data are presented as the Mean±S.E.M. percentage time spent freezing. Freezing behaviour was analyzed in a repeated measures analysis of variance (repeated measures ANOVA) with test as a within-subjects factor or by ANOVA. For repeated measures ANOVA, Mauchly's Test of Sphericity was applied. If the sphericity assumption was not met, the Greenhouse-Geisser correction was applied. *Post-hoc* planned comparisons were made using repeated measures ANOVA and the *P* value constrained by the number of comparisons made. ANOVA was applied to data from Western blot experiments. Tukey's test was then used for *post hoc* analysis to determine the sources of significance (P<0.05, P<0.01 and P<0.001).

## Results

### Contextual fear memories can be independently manipulated by context exposure

**Figure 1:**
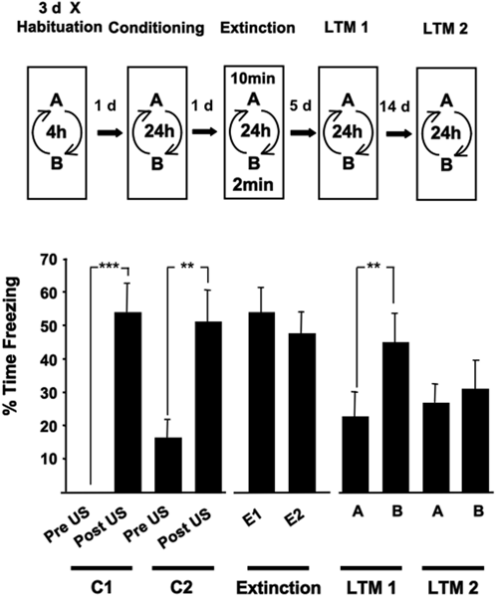
10 min exposure to a conditioned context induced the extinction of a selectively recalled fear memory. Repeated measures ANOVA revealed significant effects of the training and test phases on freezing behaviour (F _(4.329, 47.618)_ = 11.355, *P* = 0.000, ε = 0.481). Rats (n = 12) presented with a single footshock (US) in two distinct contexts (A and B) 24 hrs apart (C1 and C2) showed robust freezing behaviour in the post US period and during the first 2 min of exposure to the conditioned contexts during the two extinction training sessions (E1 and E2) two days later. All rats experienced a 2 min re-exposure to one of the conditioned contexts and a 10 min exposure to the other conditioned context in a counterbalanced manner. Rats re-exposed to the conditioned contexts 5 days later (LTM 1) showed less freezing in the context in which they experienced a 10 min exposure (A), than in the context that they had been exposed to for 2 min (B) during extinction training. At a further test 3 weeks after conditioning (LTM 2) the rats showed low levels of conditioned fear in both contexts. Results are presented as the Mean±S.E.M. * *P*<0.05, ** *P*<0.01, *** *P*<0.001.

A powerful method of measuring the effects of experimental manipulations on memory stability after recall would be to show that the manipulation selectively impacted on a recalled memory but would leave a non-recalled memory intact. Therefore, we first established a behavioural protocol by which two different hippocampal–dependent contextual fear memories (CFM) could be separately retrieved and manipulated by the duration of the exposure to the conditioned context during extinction training (Fig. 1). Firstly, rats were fear conditioned to two different contexts (A and B) by presenting a short unsignalled footshock in each of the contexts on consecutive occasions. During extinction training each rat simply received a 2 min exposure to one of the conditioned contexts and a 10 min exposure to the other conditioned context. The exposures to the contexts during the behavioural training sessions were counterbalanced across the experiment. The effect of extinction training on conditioned freezing behaviour (an index of fear memory) was also measured during two series of context re-exposure recall tests 5 and 14 days later. This protocol is illustrated in Fig. 1. Rats showed a robust conditioned freezing behaviour in the two contexts during the first 2 min of each extinction training session indicating CFM had been established for both contexts. During the recall test 5 days later (LTM1), rats characteristically showed less freezing in the context in which they had received a 10 min exposure during extinction training (A) than in the context they were exposed to for 2 min during extinction training (B, Fig. S1). We showed in a similar contextual fear conditioning procedure that a 2 min exposure to a conditioned context engaged reconsolidation processes which stabilise or maintain the fear memory for subsequent recall, as measured by high levels of conditioned freezing at all recall tests [24]. Here likewise, the maintenance of high levels of freezing in context B at LTM1 suggest reconsolidation of the fear memory for context B was induced by brief exposure to this particular context during extinction training. In the same animals, a longer 10 min exposure to context A at extinction training induced the extinction of fear memory for context A. Thus, two separate CFM could be independently modified by their context-selective recall and the conditions (duration of context re-exposure) of recall. There was no recovery of the extinguished fear memory at the second recall test, LTM2, 19 days after extinction training.

### The extinction of contextual fear memory is dependent on protein synthesis in the hippocampus

**Figure 2:**
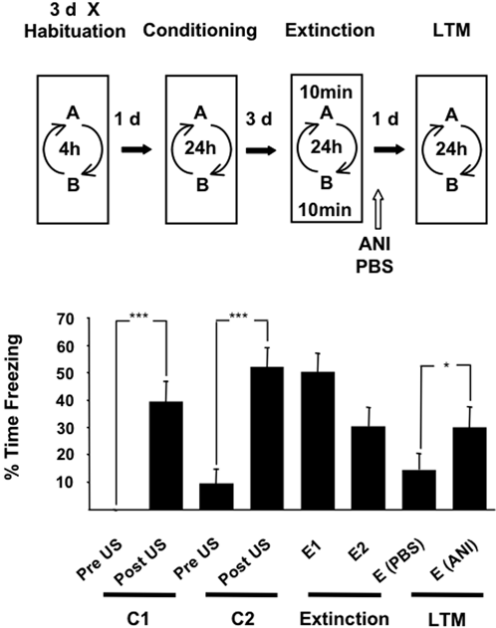
Effect of ANI infusion into the hippocampus on conditioned freezing. Rats (n = 12) were fear conditioned (C1 and C2) in two distinct contexts. ANI or PBS were infused into the hippocampus immediately after extinction training by a 10 min exposure to each of the conditioned contexts at E1 and E2 such that each rat received ANI associated with one context and PBS with the other context in a counterbalanced fashion. ANI prevented the extinction of a selectively recalled fear memory because conditioned freezing measured at LTM in the context associated with ANI (E(ANI)) was greater than conditioned fear measured in the PBS associated context (E (PBS)). Results are presented as the Mean±S.E.M. *p<0.05, ***p<0.001.

To determine whether the extinction of contextual fear memory was dependent on the hippocampus, and more specifically required protein synthesis in this brain region, we used a similar behavioural training procedure to the previous experiment except that during extinction training each rat received a 10 min exposure to both of the conditioned contexts. The protein synthesis inhibitor, ANI, and PBS were infused into the hippocampus immediately after extinction training sessions E1 and E2 (Fig. 2). Infusions were administered in a counterbalanced fashion such that half the rats received ANI at E1 and PBS at E2, and vice versa for the remaining rats. There were significant effects of the training and test phases on freezing behaviour (F _(3.130, 40.691)_ = 11.990, *P* = 0.000, ε = 0.447, repeated measures ANOVA). These were characterised by freezing behaviour in the conditioning context only after footshock presentation (C1 and C2), and by conditioned freezing behaviour during the first 2 min of the two extinction trials (E1 and E2). Rats froze significantly less in the context paired with PBS infusions during extinction than in the context paired with ANI during the recall test (LTM). Thus, ANI attenuates the apparent loss of freezing behaviour produced by a prolonged 10 min exposure to a fear-conditioned context. This indicates that extinction of CFM was dependent on protein synthesis in the hippocampus. The within-subjects design of this experiment again demonstrates that two different fear memories can be independently modulated.

### Extinction is correlated with increased proBDNF and decreased Arc/Arg3.1 in CA1

**Figure 3:**
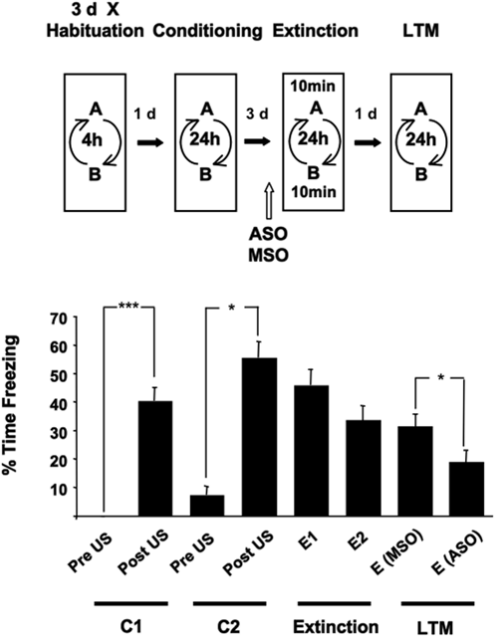
Effect of BDNF ASO infusion into the hippocampus on conditioned freezing. BDNF ASO and BDNF MSO were infused into the hippocampus after extinction training by a 10 min exposure to two fear conditioned contexts (A and B) at E1 and E2 in a counterbalanced fashion (n = 23). BDNF ASO enhanced the extinction of a selectively recalled contextual fear memory since less conditioned freezing was seen during LTM tests in the context associated with BDNF ASO infusion (E(AS)) than the context associated with BDNF MSO infusion (E(MSO)). Results are presented as the Mean±S.E.M.. Data for the first 2 min of extinction training during E1 and E2 is shown. (F _(5.401, 113.461)_ = 31.319, *P* = 0.000, ε = 0.772, repeated measures ANOVA). * *P*<0.05, ** *P*<0.01, *** *P*<0.001.

To assess whether the extinction of contextual fear memory required BDNF in the hippocampus, ASO targeting BDNF mRNA was infused into the hippocampus 90 min prior to extinction training in one of the two conditioning contexts. Control MSO was infused before exposure to the other conditioned context (Fig. 3). During the subsequent LTM recall test, conditioned freezing behaviour was lower in the context paired with ASO infusions than in the context paired with MSO infusions. In addition, less freezing was seen in the ASO context, but not MSO context, than during extinction training. The infusion of ASO had no effect on the freezing behaviour during the extinction training sessions at E1 and E2 (Extinction×ASO×Freezing, F _(1, 41)_ = 0.313, *P* = 0.579, ε = 1.000 repeated measures ANOVA), demonstrating the ASO infusions do not alter the acquisition of extinction nor change hippocampal processing non-specifically. One interpretation of these data is that MSO specifically prevents the extinction of contextual fear memory. However, this is unlikely as a NCBI BLAST search revealed that the MSO sequence does not show any homology with existing nucleotide sequences and would not act to prevent translation of any known transcript. We suggest that ASO targeting BDNF in the hippocampus promotes the extinction of contextual fear memory.

**Figure 4:**
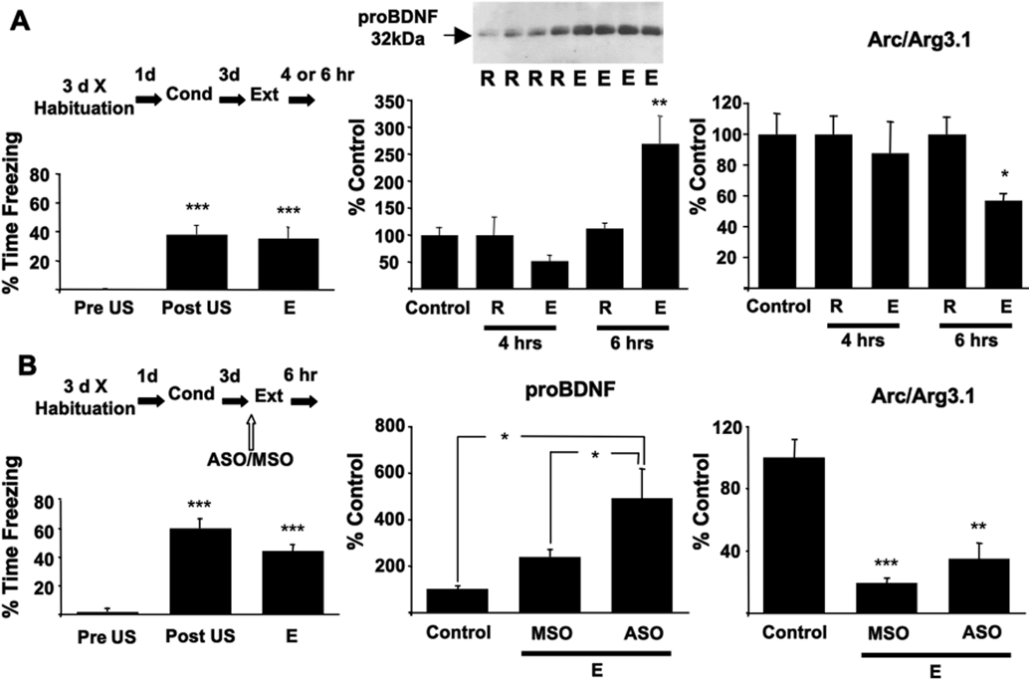
Extinction training-induced changes in proBDNF and Arc/Arg3.1 protein in the CA1 of hippocampus. (a) Rats showed robust conditioned freezing during the first two min re-exposure to the training context (E) 3d after a single fear conditioning trial (C). n = 20 at C, and n = 16 at E. Following recall there was a change in proBDNF in the CA1 (F _(4,14)_ = 8.961, *P* = 0.000, ANOVA). ProBDNF levels more than doubled in CA1 6 hrs after a 10 min exposure to the conditioned context (E). No changes were seen after a 2 min exposure to the fear-conditioned context (R). Arc/Arg3.1 protein in CA1 decreased 6 hrs after a 10 min exposure (E) but not following a 2 min exposure (R) to the conditioned context. (b) High levels of conditioned freezing were seen in rats administered intrahippocampal infusions of ASO and MSO 90 min before extinction training. There was no difference in the levels of freezing between the ASO and MSO administered rats at E (F _(1, 7)_ = 4.202, *P* = 0.080, ANOVA). However, proBDNF levels in CA1 were altered after extinction (F _(2, 9)_ = 6.974, *P* = 0.015, ANOVA) and were greater in the ASO administered when compared to control and MSO administered rats 6 hours after extinction. In the same rats, protein levels of Arc/Arg3.1 were also regulated in CA1 (F _(2, 9)_ = 23.742, *P*>0.000, ANOVA), but were decreased in both MSO and ASO groups. Rats in the control group were fear conditioned at C, but were killed 3 d later. n = 4 for all groups in Western blot measurements. Results are the Mean±S.E.M. *P<0.05, **P<0.01, ***P<0.001 compared to control unless otherwise marked.

To further elucidate the role of BDNF in the extinction of contextual fear, the levels of the BDNF-precursor, proBDNF and Arc/Arg3.1 were measured in extracts of CA1 after extinction training (Fig. 4a). Arc/Arg3.1 is a BDNF-regulated gene [29], [30], [31] that is necessary for both LTM and LTP [32], [33]. We previously showed that intrahippocampal infusions of ASO targeting BDNF prevented the increase in Arc/Arg3.1 protein associated with contextual fear conditioning and CFM [24]. We also showed that the inhibitory effects of the ASO on function were rescued by mBDNF. Recent evidence also shows that mBDNF-induced LTP in the hippocampus is mediated by Arc/Arg3.1 synthesis [34]. Together these data demonstrate a requirement for mBDNF regulated Arc/Arg3.1 in the hippocampus for the consolidation of CFM and enduring forms of plasticity. As such, measuring Arc/Arg3.1 levels in the hippocampus represents bioassay of mBDNF activity associated with CFM processing.

Here, 48 hours after rats were conditioned to one context they underwent recall under conditions that induce either reconsolidation (2 min exposure) or extinction (10 min exposure) of CFM. A 250% increase in proBDNF in CA1 was measured 6 hours after recall conditions that produce extinction. The increase in proBDNF levels was accompanied by a 50% decrease in Arc/Arg3.1. There were no changes in proBDNF and Arc/Arg3.1, 4 or 6 hours after recall in the dentate gyrus (dg, *data not shown*). This agrees with cellular and molecular studies at the subregional level that show a selective role for the CA1 activity after the acquisition and retrieval of CFM [35], [36], [37]. Intrahippocampal infusions of ASO prior to a 10 min extinction trial significantly increased the levels of proBDNF protein in the CA1 6 hrs after extinction but had no effect on the decrease in Arc/Arg3.1 (Fig. 4b).

These results show a direct correlation between the levels of proBDNF in the hippocampus after extinction and the magnitude of extinction of contextual fear memory. Moreover, the results also show an inverse correlation between levels of the uncleaved precursor of BDNF, proBDNF and the *activity* of mature BDNF in the CA1 following the extinction of CFM suggesting that extinction of long-term memories is mediated by the processing of BDNF in CA1.

### Extinction is correlated with Decreased Processing of BDNF in CA1

**Figure 5:**
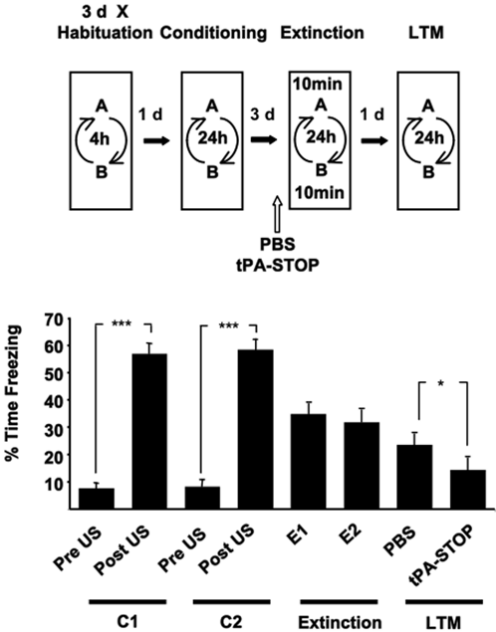
Infusions of tPA-STOP into the hippocampus potentiate extinction of contextual fear memory. Rats (n = 11) received two 10 min extinction-training trials (E1 and E2 24 hr apart) 3 days after contextual fear conditioning in two distinct contexts (A and B). Prior to E1 they either received tPA-STOP (n = 6) or PBS (n = 5). The same rats received these compounds prior to E2 such that each rat was infused with tPA-STOP in one of the two conditioned contexts and vehicle in the other during extinction. The rats showed more conditioned freezing in the context associated with the vehicle PBS infusions than in the extinction context associated with tPA-STOP infusions during subsequent long-term memory recall tests (LTM). Results are presented as the Mean±S.E.M. Data for the first 2 min of extinction training during E1 and E2 is shown. (F _(3.688, 36.88)_ = 35.063, *P* = 0.000, ε = 0.0.526, repeated measures ANOVA). **P<0.01, ***P<0.001.

To test the hypothesis that the extinction of fear memories is mediated by the proteolytic processing of proBDNF, the synthetic competitive inhibitor of tPA, tPA-STOP (2,7-bis-4(amidino-benzylidene)-cycloheptanone-1-dihydochloride) [38] was infused into the hippocampus prior to extinction training. We predicted that preventing the cleavage of proBDNF to mBDNF with tPA-STOP during extinction training would promote the extinction of contextual fear memory. Again we conditioned individual rats so that they formed two independent CFM's. The extinction of one CFM occurred after intrahippocampal infusions of tPA-STOP (Fig. 5). There was no effect of tPA-STOP on the conditioned freezing behaviour during the two extinction training phases (comparing the behaviour between the first and last two minutes of E1 with the same epochs in E2) of the experiment (Freezing×Epoch×tPA-STOP, F _(1, 18)_ = 2.165, *P* = 0.158, ANOVA; Freezing×tPA-STOP interactions, F _(1, 18)_ = 0.004, *P*>0.950, ANOVA). Thus suggesting that tPA-STOP has no effect on the performance during extinction training and the acquisition of extinction. However during the LTM recall tests, conditioned freezing was significantly less in the tPA-STOP-associated extinction context than in the vehicle-associated context. These results show tPA-STOP potentiated the extinction of CFM. This effect of tPA-STOP cannot be attributed to a general amnesic of the tPA inhibitor because all rats were administered tPA-STOP, but its effects on CFM were limited to the memory recalled during extinction. Furthermore, there were no affects on long-term hippocampal function because there was no evidence of a spontaneous recovery of the memory when measured one week later and ability to support a new CFM was not compromised when rats were subsequently reconditioned (*Supplementary Information,* *Fig. S2*).

**Figure 6:**
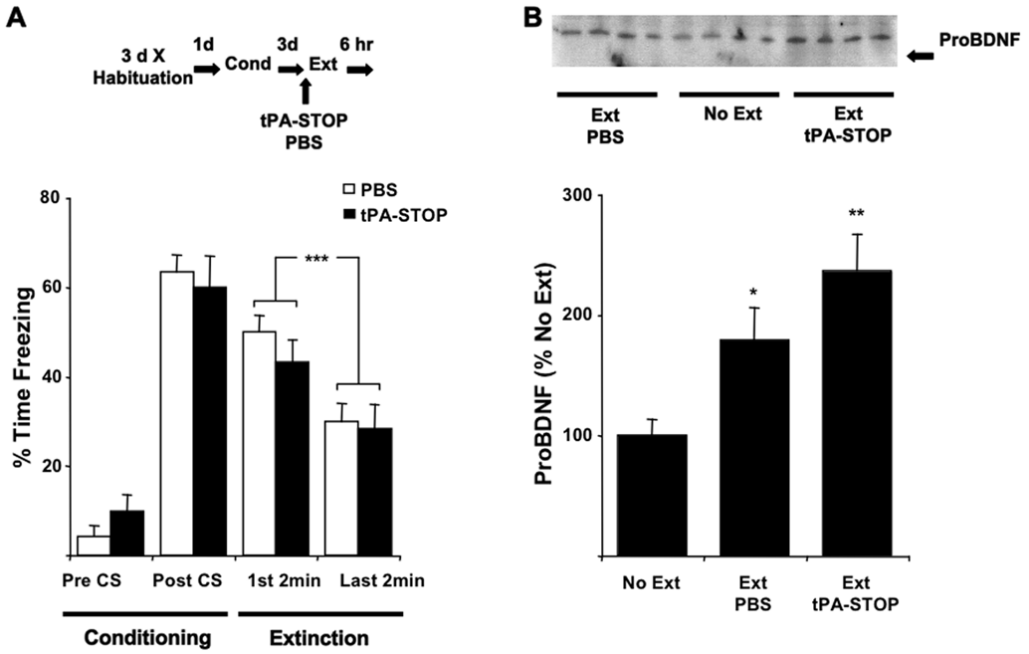
Infusions of tPA-STOP into the hippocampus potentiate the proBDNF levels in CA1 after extinction. (a) Rats (n = 18) received a single conditioning trial. 90 min prior to extinction, 3 days later, they either received tPA-STOP (n = 6) or PBS (n = 6). tPA-STOP had no effect on the decrement in the fear response measured between the first and last two minutes of extinction training. (b) There was a significant effect of tPA-STOP on CA1 proBDNF after extinction (F (2,15) = 8.003, P = 0.004, ANOVA, Fig. 6b) in the same conditioned rats 6 hr after extinction. Results are presented as the Mean±S.E.M. *P<0.01, **P<0.001 compared to No Ext group.

In addition to being an upstream activator of proBDNF cleavage, tPA has other molecular targets that may underlie the effect of tPA-STOP on extinction we report. For example, the tPA-mediated degradation of the NR1 subunit of the NMDA receptor and the extracellular matrix, as well as tPAs interaction with the low-density lipoprotein receptor related protein have been reported to influence plasticity processes in the brain [39], [40], [41], [42]. To assess whether tPA-STOP regulates proBDNF processing in extinction, proBDNF and Arc/Arg3.1 levels in CA1 were measured after extinction training (10 min recall test) following the intrahippocampal administration of tPA-STOP. Although there was a significant effect of conditioning and extinction (TEST PHASE) on freezing behaviour (F (2.079,20.788) = 45.965, P = 0.000, ε = 0.693, repeated measures ANOVA), there was no tPA-STOP X TEST PHASE interaction (F (2.079,20.788) = 0.509, P = 0.679, ε = 0.693, repeated measures ANOVA, Fig. 6a). tPA-STOP had no effect on the decrement in the fear response measured between the first and last two minutes of extinction training (“within-session” extinction of freezing). This again illustrates that tPA-STOP has no effect on the performance during extinction training, or on the acquisition of extinction. There was a significant effect of tPA-STOP on CA1 proBDNF after extinction (Fig. 6b). This was characterised by an increase in levels compared to the No Ext control rats that was further increased by tPA-STOP. Hence tPA activity regulates proBDNF levels in CA1 during the extinction of CFM. Arc/Arg3.1 was unaffected by tPA-STOP after extinction (F (2,15) = 0.562, P = 0.581, ANOVA; *Levels (% No Ext); No Ext = 100±22.7, Ext-PBS = 71.3±18.4, Ext-tPA-STOP = 81.4±16.5*). The increased ratio of proBDNF: Arc/Arg3.1 in CA1 under conditions of extinction further indicates decreased proBDNF processing by tPA after extinction.

Reconsolidation of CFM was not associated with the regulation of hippocampal BDNF or Arc/Arg3.1 levels (Fig. 4), nor requires BDNF [24]. Therefore, the long-term loss of freezing responses associated with tPA-STOP administration at recall is likely to directly reflect the impact on BDNF-mediated cellular signalling mechanisms underlying extinction rather than reconsolidation. This concurs with studies that suggest BDNF signalling is necessary for the extinction of fear memory [43], [44], but crucially indicates a role for the processing of proBDNF. Common to a number of studies that show that the extent of memory reactivation greatly influences extinction induction [45], [46], we also show that increasing the duration of context reexposure from 2 min to 10 min results in persistent, reduced conditioned freezing behaviour. It is possible that the extent of proBDNF cleavage is precisely controlled by the conditions of memory recall and that higher levels of proBDNF favour extinction as the dominant trace controlling behaviour after recall by engaging specific downstream cellular events.

### tPA-STOP attenuates consolidation

**Figure 7:**
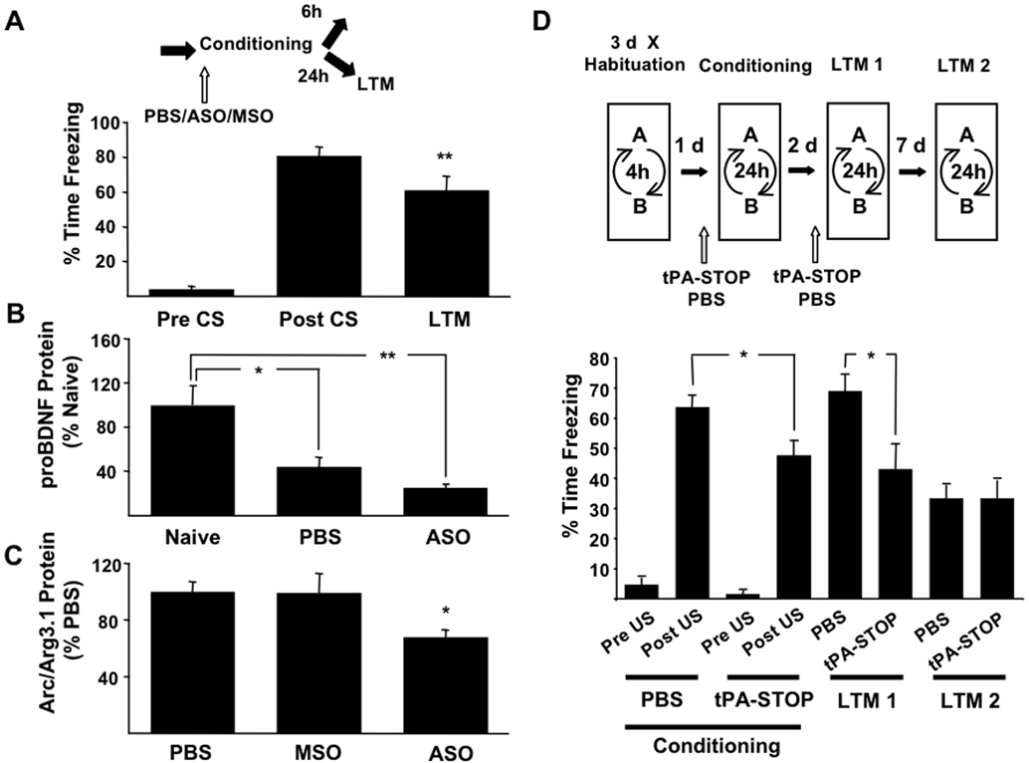
Fear conditioning-induced changes in proBDNF and Arc/Arg3.1 protein in the CA1 of hippocampus. (a) Rats showed conditioned freezing at LTM test 24 hrs after a single conditioning trial. n = 15 at C, and n = 3 at LTM. (b) ProBDNF decreased by half in the CA1 6 hrs after conditioning in the PBS-infused hippocampus. This was further reduced by BDNF ASO (ASO) infusions into the hippocampus prior to conditioning (F _(2, 9)_ = 12.894, *P* = 0.002, ANOVA). (c) Arc/Arg3.1 protein in CA1 was selectively decreased in rats receiving BDNF ASO, but not PBS or BDNF MSO (MSO) infusions prior to conditioning. n = 4 (d) A separate group of rats (n = 11) were fear conditioned to two contexts (A and B), they received intrahippocampal infusions of tPA-STOP 90 m in before conditioning in one of the contexts and vehicle prior to training in the other. Half the rats received tPA-STOP or PBS prior to the recall test LTM 1 to determine the effect of tPA-STOP of conditioned freezing. The rats showed less post US freezing behaviour during conditioning with pretraining tPA-STOP infusions and less conditioned freezing in the drug associated context during LTM 1 compared to the control PBS context. Results are the Mean±S.E.M. (F _(3.022, 30.221)_ = 28.352, *P* = 0.000, ε = 0.432, repeated measures ANOVA). ** *P*<0.01 compared to Pre CS, * *P*<0.01 compared to PBS and MSO groups for unmarked comparisons.

mBDNF activity in the hippocampus is a prerequisite for the consolidation of CFM because ASO-mediated amnesia could be completely rescued by the concurrent administration of the proteolytically cleaved mBDNF protein [24]. The increased expression of Arc/Arg3.1 also suggested the activity of mBDNF was upregulated in CA1 following acquisition. Here we show that levels of proBDNF were also regulated during the consolidation of contextual fear memory (Fig. 7). Planned *post hoc* analyses revealed a 60% decrease in CA1 proBDNF in MSO-infused hippocampus 6 hours after contextual fear conditioning that were further reduced in ASO-infused hippocampus (Fig. 7b). Intrahippocampal infusion of ASO targeting BDNF mRNA before conditioning reduced the levels of Arc/Arg3.1 protein in the CA1 6 hours later compared to Arc/Arg3.1 measured in vehicle (PBS) and MSO infused control groups (Fig. 7c). There was no difference between Arc/Arg3.1 in CA1 in PBS and MSO groups further emphasising that the MSO used in our studies is biologically inactive. Thus we show that the levels of proBDNF decreased and the *activity* of mature BDNF increased in CA1 after fear conditioning. In addition, we also show amnesia-promoting ASO administration down-regulated both proBDNF and Arc/Arg3.1. These data suggest a correlation between the increased processing of proBDNF in CA1 in the formation or stabilisation of CFM.

We next investigated whether the proteolytic processing of proBDNF was causal in the formation of long-term fear memories. Rats were fear conditioned in two distinct contexts. They received intrahippocampal infusions of tPA-STOP before conditioning in one context and PBS vehicle control prior to conditioning in the other context (Fig. 7d). Half the rats also received tPA-STOP prior to the recall test at LTM1. During conditioning rats showed less post US freezing behaviour after tPA-STOP than vehicle infusion. Pre-recall test tPA-STOP had no effect on freezing behaviour during LTM1 demonstrating that tPA-STOP did not affect performance *per se* (F = 0.122 (1,9) P = 0.735, ANOVA). The rats also showed less conditioned freezing in the tPA-STOP-associated context than the vehicle-associated context during recall at LTM1, which did not recover 7 days later. Together these data demonstate that the acquisition of CFM is associated with increased proBDNF processing in the hippocampus.

Although the consolidation of CFM is critically dependent on the mBDNF in the hippocampus, a role for proBDNF in consolidation was not previously ruled out [24]. This study shows that acquisition of CFM was correlated with a decrease in proBDNF levels in CA1. One interpretation is that decreased proBDNF-mediated signalling is also a necessary requirement for the formation of LTM. If proBDNF mediated cellular processes normally opposed consolidation, then reductions in proBDNF in the absence of changes in baseline mBDNF activity would be permissive for consolidation. However, here we show the opposite effect; infusions of ASO that prevent consolidation [24]. further reduced proBDNF levels after conditioning, while Arc/Arg3.1 levels were normalised. Thus, results from our studies are entirely consistent with a selective role for mBDNF-mediated processes in acquisition of long-term memory.

## Discussion

This study provides novel insights into the molecular processes during the acquisition of long-term fear memories and those processes triggered by their selective recall. We show that reduced proteolysis of proBDNF in the hippocampus is a key regulator in protein synthesis-dependent extinction of CFM. Critically, increasing endogenous proBDNF and reducing mBDNF levels in the CA1 either with BDNF ASO or tPA-STOP, promoted extinction. Conversely, the acquisition of CFM was correlated with increased proteolytic processing of proBDNF. The demonstration of a role for BDNF in the acquisition of LTM has not been previously dissected in more chronic transgenic or pharmacological animal models. We have previously shown that consolidation but not reconsolidation of CFM is dependent on hippocampal BDNF [24]. Here we also show that conditions of recall that initiate the reconsolidation are not correlated with a change in proBDNF levels and mBDNF activity in the CA1. Therefore, the processing of BDNF was associated with the acquisition of new information and the updating of information about a salient stimulus that mediate changes in behaviour. These data generate a complete hypothesis for BDNF-associated signalling in the currently described component processes of LTM. Thus, BDNF regulates the acquisition, consolidation and extinction of fear memory, but not reconsolidation. In addition, the tPA-mediated proteolysis of proBDNF promotes new learning but opposes the extinction of established memory.

The competition between extinction and reconsolidation are governed by the precise conditions of memory reactivation [45], [46]. Here we show that proBDNF cleavage is selectively inhibited under conditions of recall that favour extinction (a prolonged 10 min exposure to the context CS), but not those that promote reconsolidation (a 2 min CS presentation). This clearly demonstrates the fine control of cellular responses by ongoing experience. The differential control of the proteolysis of proBDNF by salient environmental stimuli in new learning and by learning anew after recall, also indicates the integration of new and past experience at the molecular level. Determining the molecular or cellular mechanism necessary for integrating experience will be an important endeavour. That an inhibitor of BDNF processing, tPA-STOP, can attenuate new learning but potentiate extinction, further emphasises a central role for the integration of new and past experience at the molecular level in determining future behavioral responses.

This study indicates that secretion and processing of proBDNF in the adult hippocampus occurs as a consequence of memory formation. Firstly, we detected a BDNF-immunopositive signal at 35kDa (the molecular size of proBDNF) in CA1 that is specifically altered by regional infusions of ASO BDNF. This suggests that the signal is derived from the *Bdnf* gene. Indeed studies of CNS neurons transfected with *Bdnf* cDNA suggest that proneurotrophins account for a significant amount of the total neutotrophins secreted extracellularly [47], [48]. Secondly, the levels of proBDNF were regulated during consolidation and extinction. Thirdly, we showed that regional administration of tPA-STOP, an upstream inhibitor of the extracellular proteolysis of precursor BDNF [38], attenuated the processing of proBDNF in CA1. Significantly, we showed that altering the ratio of precursor to mature BDNF levels with tPA-STOP and ASO BDNF has important functional consequences for LTM. Our data concurs with other studies that have shown that several forms of long-term plasticity in the adult hippocampus were correlated with changes in BDNF processing by the extracellular protease, tPA [28], [49], [50]. It should be noted that a recent study of endogenous BDNF processing in primary cell culture has shown little, if any, proBDNF is stored and secreted from hippocampal neurons [51]. However, the failure to detect proBDNF secreted from neurons derived from embryonic tissue, in which BDNF expression is comparatively low, may consequently reflect different dynamic levels of neurortrophin transport, release and processing mechanisms to those occurring in adult neurons [26].

The mechanism by which BDNF ASO potentiated the increase in proBDNF levels after extinction is unknown. It is possible that these effects may be caused by non-selective off-target, non-sequence specific effects of infusing oligonucleotides into the brain, such as the direct interaction with cellular protein or by activating immune responses [52]. However, this explanation is unlikely because the manipulation of hippocampal proBDNF protein levels and extinction of fear memory were selective for ASO and not MSO. Furthermore, the ASO and MSO had no effect on the levels of β-Actin, the not regulated reference protein used in the above experiments (*data not shown*). We have also previously reported effects of the BDNF ASO, but not Zif268 ASO or MSO sequences on mBDNF activity in the CA1 and the consolidation of CFM [24]. Therefore the behavioural and cellular responses to ASO are selective and are related to the targeted mRNA sequence.

Protein noncoding antisense transcripts expressed from human BDNF gene locus have been identified and may function to regulate BDNF gene expression *in vivo* [53]. Therefore, it is possible that exogenous ASO infusions may interfere with the mechanism of action of endogenous antisense-BDNF to alter BDNF levels in the hippocampus. However, this explanation for the BDNF-ASO potentiated increase in proBDNF we observed is doubtful because in contrast with the human BDNF gene locus, rodent *Bdnf* gene loci do not encode antisense-BDNF mRNA transcripts [53], [54].

Evidence from several elegant studies have suggested that opposing cellular actions of mBDNF and proBDNF mediate synaptic plasticity [55]. Namely, the cleavage of proBDNF to mBDNF by tPA is essential for LTP in the hippocampus [28]. Whilst proBDNF-mediated signalling facilitates LTD in the hippocampus via the activation of the p75 neurotrophin receptor [27]. Our evidence that hippocampal-dependent extinction is mediated by an increased proBDNF/mBDNF ratio further suggests that that the synaptic and molecular events underlying extinction closely resembles LTD [18], [20], [21], [22], [23], [56]. Our studies also show dissociable roles for mBDNF and proBDNF in the consolidation and extinction of hippocampal-dependent fear conditioning. The close correlation between the control of synaptic memory and the expression of CFM and extinction by different translational variants of BDNF, may indicate that different forms of synaptic plasticity models distinct memory processes. The precise cellular mechanism that controls the processing of BDNF by tPA required for the acquisition and extinction of long-term memory remains to be determined.

The illustration that the proteolysis of proBDNF is a key regulator of two-hippocampal dependent memory processes clearly demonstrates the significant role that post-translational protein modifications (PTM) play in LTM. Recently, a mechanistic model has proposed that PTM of synaptic proteins, maintained by endogenous brain activity, play an instructive role for LTM [2]. A consequent prediction in this model is that manipulations that alter the PTM of proteins crucial for maintaining LTM cause the loss of the memory. This has recently been shown for PKMζ [57]. The model has some face validity for our data here because increased proteolysis of proBDNF was associated with the formation of LTM, while decreased processing was associated with the apparent loss (extinction) of LTM. However, we show that experimental interventions that alter the processing of BDNF are selective for the recently acquired or recalled memories, the so-called *active* memory [58]. Non-recalled, *inactive* memories were unaffected. This implies that there is a time-limited role for PTM of BDNF in LTM. In addition, since ASO targeting BDNF has no effect memory or BDNF processing after some conditions of recall (reconsolidation) [24], this suggests that the on-going maintenance of CFM is not dependent on BDNF, or the post-translational state of BDNF. This implies that BDNF is permissive for LTM by initiating the PTM of other synaptic proteins that have an instructive role in LTM, via the activation of specific signalling pathways. Future experiments are required to address this possibility.

The requirement of BDNF dependent-processing for the extinction but not reconsolidation of LTM after recall suggests that drug or other interventions that directly target the PTM of BDNF, or the downstream signalling pathways of BDNF variants, potentially offers the therapeutic control of pathological memory in humans. For example, the memories that are considered to underlie phobia, post-traumatic stress disorder and drug addiction. Targeting BDNF may be particularly useful because only recalled, active, memories appear to be sensitive to manipulations that regulate with the cleavage of proBDNF to mBDNF. This has the advantage of leaving non-recalled memories intact. Furthermore, inhibiting the processing of proBDNF at recall would additionally prevent the acquisition of new memories that may be associated with the therapeutic environment and which may trigger the re-emergence of the memory by the process of renewal once away from the extinction environment [6], or cause the sensitization (augmentation) of the pathological memory [59], [60].

## Supporting Information

Figure S1 Two separate fear memories can be independently modulated by extinction training. Five days after extinction training lower levels of conditioned freezing were measured during a recall test in the context the rats has been exposed to for 10 min during extinction training, E, than in the context that had been associated with a 2 min exposure, R, irrespective of whether context A (E(A)) or context B (E(B)) was the 10 min extinction context. Two-way repeated measures ANOVA of the freezing behaviour during T1 revealed an Extinction Training X Context interaction (F = 12.476 (1,10), p = 0.005), but no significant effect of Context (F = 0.024 (1,10), p = 0.897). Results are presented as the Means.
(6.01 MB TIF)

Figure S2 Infusions of tPA-STOP into the hippocampus potentiate extinction of contextual fear memory and have no long-term effect on hippocampal function. As described previously (Fig. 5), rats (n = 11) received two 10 min extinction-training trials (E1 and E2 24 hr apart) 3 days after contextual fear conditioning in two distinct contexts (A and B). Prior to E1 they either received tPA-STOP (n = 6) or PBS (n = 5). The same rats received these compounds prior to E2 such that each rat was infused with tPA-STOP in one of the two conditioned contexts and vehicle in the other during extinction. Rats showed more conditioned freezing in the context associated with the vehicle PBS infusions than in the extinction context associated with tPA-STOP infusions during long-term memory recall test (LTM 1) 1 day after extinction. Additionally, this data showed that there was no spontaneous recovery of the fear memory measured at a subsequent recall test 7 days later. All rats were re-conditioned in one context (A or B). A recall test was performed in both the contexts 1-2 days later (LTM3). At LTM3 rats showed significantly higher levels of conditioned freezing in the reconditioned context (C3) than in the context not associated with reconditioning (no C3). This indicates that (i) tPA-STOP has no long-term affect on hippocampal function because the rats can support anew a contextual fear memory for a specific context. (ii) There is no reinstatement of the extinguished fear memory by exposure to the US (Rescorla and Heth, 1975) because freezing behaviour was specific to the context in which the animals were reconditioned. These results demonstrate that tPA-STOP infused into the hippocampus selectively attenuates the extinction of contextual fear memory. Results are presented as the Mean±S.E.M. Data for the first 2 min of extinction training during E1 and E2 is shown. (F (4.997, 49.970) = 32.047, P = 0.000, ε = 0.454, RM ANOVA). *P<0.05, **P<0.01. Rescorla RA, Heth CD (1975) Reinstatement of fear to an extinguished conditioned stimulus. J Exp Psychol Anim Behav Process 1:88-96.
(6.01 MB TIF)
