# Supplementary figures and images for: Proteolysis of proBDNF Is a Key Regulator in the Formation of Memory
Source: PLoS One. 2008 Sep 24;3(9):e3248. doi: 10.1371/journal.pone.0003248 (PMC2532744; doi:10.1371/journal.pone.0003248)

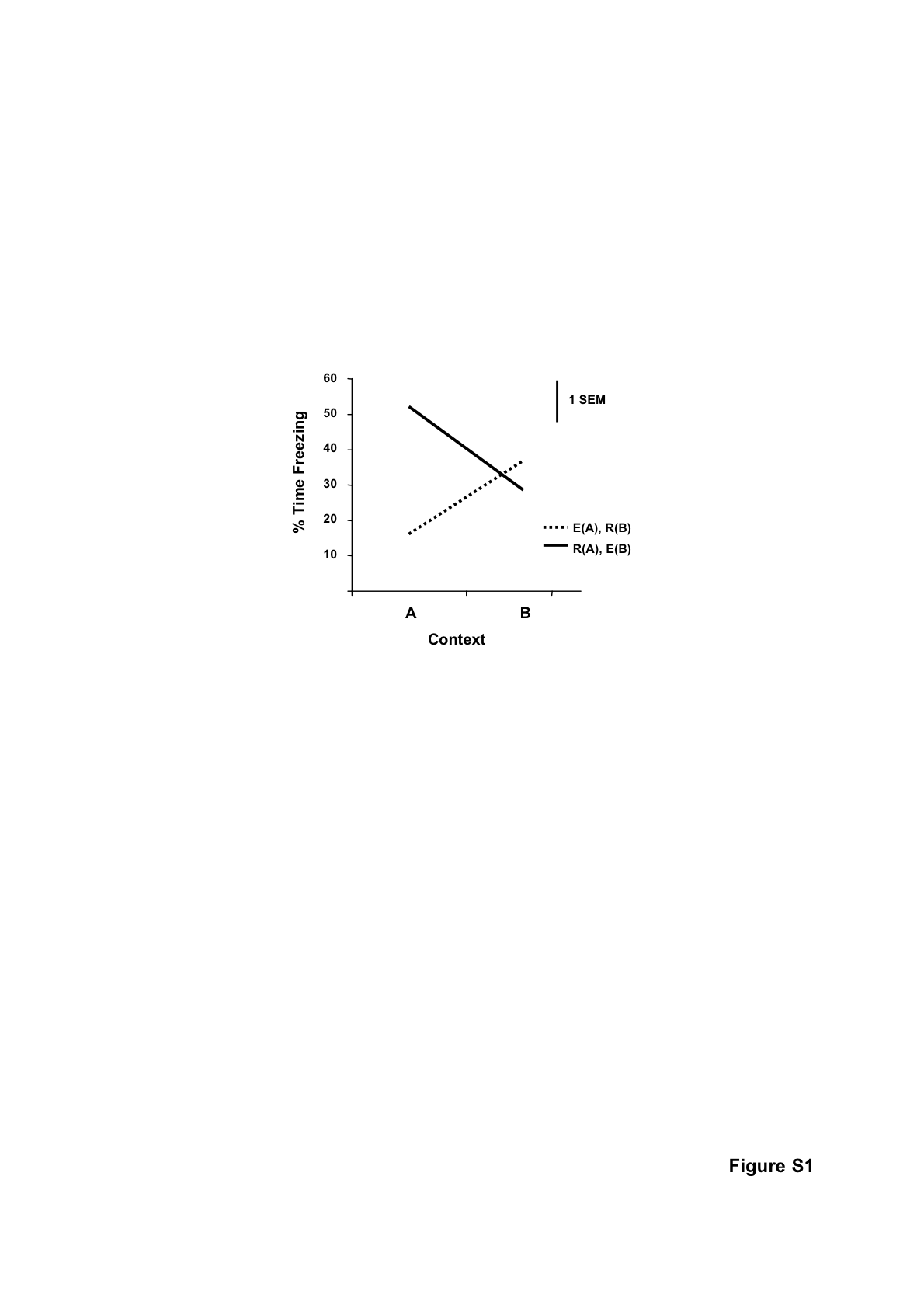

Supplement: Figure S1 — Two separate fear memories can be independently modulated by extinction training. Five days after extinction training lower levels of conditioned freezing were measured during a recall test in the context the rats has been exposed to for 10 min during extinction training, E, than in the context that had been associated with a 2 min exposure, R, irrespective of whether context A (E(A)) or context B (E(B)) was the 10 min extinction context. Two-way repeated measures ANOVA of the freezing behaviour during T1 revealed an Extinction Training X Context interaction (F = 12.476 (1,10), p = 0.005), but no significant effect of Context (F = 0.024 (1,10), p = 0.897). Results are presented as the Means. (6.01 MB TIF) [file pone.0003248.s001.tif]

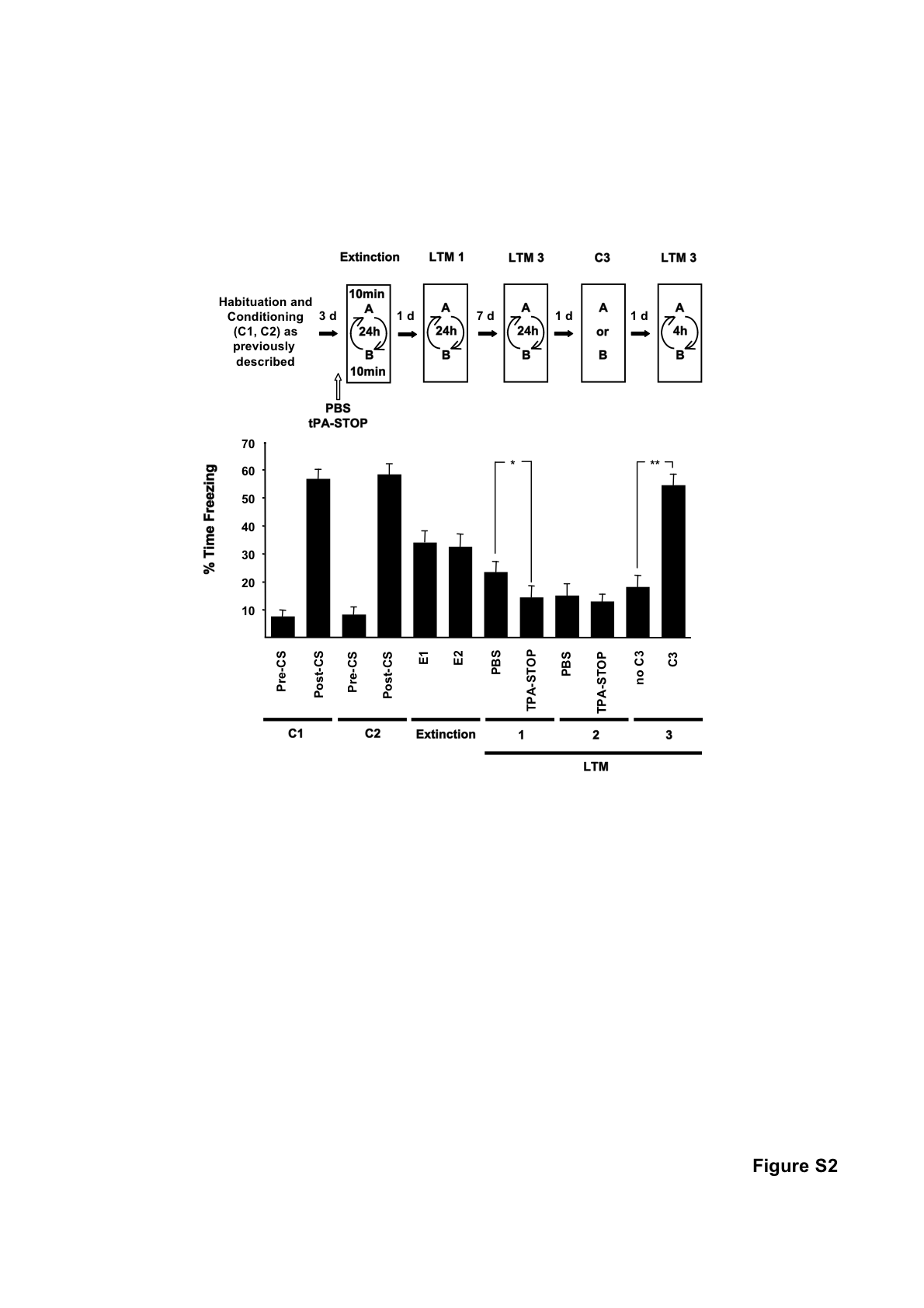

Supplement: Figure S2 — Infusions of tPA-STOP into the hippocampus potentiate extinction of contextual fear memory and have no long-term effect on hippocampal function. As described previously (Fig. 5), rats (n = 11) received two 10 min extinction-training trials (E1 and E2 24 hr apart) 3 days after contextual fear conditioning in two distinct contexts (A and B). Prior to E1 they either received tPA-STOP (n = 6) or PBS (n = 5). The same rats received these compounds prior to E2 such that each rat was infused with tPA-STOP in one of the two conditioned contexts and vehicle in the other during extinction. Rats showed more conditioned freezing in the context associated with the vehicle PBS infusions than in the extinction context associated with tPA-STOP infusions during long-term memory recall test (LTM 1) 1 day after extinction. Additionally, this data showed that there was no spontaneous recovery of the fear memory measured at a subsequent recall test 7 days later. All rats were re-conditioned in one context (A or B). A recall test was performed in both the contexts 1-2 days later (LTM3). At LTM3 rats showed significantly higher levels of conditioned freezing in the reconditioned context (C3) than in the context not associated with reconditioning (no C3). This indicates that (i) tPA-STOP has no long-term affect on hippocampal function because the rats can support anew a contextual fear memory for a specific context. (ii) There is no reinstatement of the extinguished fear memory by exposure to the US (Rescorla and Heth, 1975) because freezing behaviour was specific to the context in which the animals were reconditioned. These results demonstrate that tPA-STOP infused into the hippocampus selectively attenuates the extinction of contextual fear memory. Results are presented as the Mean±S.E.M. Data for the first 2 min of extinction training during E1 and E2 is shown. (F (4.997, 49.970) = 32.047, P = 0.000, ε = 0.454, RM ANOVA). *P<0.05, **P<0.01. Rescorla RA, Heth CD (1975) Reinstatement of f [file pone.0003248.s002.tif]
